# Comparison of Volatile Compounds among Four Types of Teas Analyzed Using Gas Chromatography–Ion Mobility Spectrometry

**DOI:** 10.3390/foods13132043

**Published:** 2024-06-27

**Authors:** Li Guo, Chenxi Xie, Feng Zhao, Yue Zhang, Zhi Lin

**Affiliations:** 1 Tea Research Institute, Chinese Academy of Agricultural Sciences, Hangzhou 310008, China; xiechenxin@tricaas.com (C.X.); zhangyue@tricaas.com (Y.Z.); linz@tricaas.com (Z.L.); 2 Graduate School of Chinese Academy of Agricultural Sciences, Beijing 100081, China

**Keywords:** green tea, yellow tea, white tea, black tea, *Camellia sinensis* (L.) Kuntze ‘Zhongcha 111’, GC-IMS, volatile compound

## Abstract

Gas chromatography–ion mobility spectrometry (GC-IMS) is a smart method that has been applied to determine the volatile compounds in Chinese teas, but its use in comparing the volatile compounds of different types of tea has not been mentioned. In this study, the volatile compounds found in four types of samples (green, yellow, white, and black teas) made with fresh leaves of *Camellia sinensis* (L.) Kuntze ‘Zhongcha 111’ were analyzed using GC-IMS. The results showed that 93 volatile compounds were identified from our tea samples and that the average volume of aldehydes was higher than that for other compounds, especially in white tea. The different samples were successfully categorized using multivariate statistical analysis. Using partial least squares discriminant analysis (PLS-DA), we found 15 key compounds, including four differential components: (E)-2-hexenal, 2-furanmethanethio, 2-hexanol, and 1-octene. There were 29 common components, and their total content reached 386.0 μg/g. Moreover, the 3-methyl-2-butenal and dimethyl disulfide detected in the four samples were also differential compounds, varying according to the manufacturing technology. Thus, this study demonstrates that different types of teas can be discriminated easily using GC-IMS and that this is helpful to shorten the time for improving tea quality and developing new products.

## 1. Introduction

Tea is known to be one of the most popular beverages in the world and it is thought to be the most widely consumed non-alcoholic drink after water. It is divided into six types, green, yellow, white, oolong, black, and dark tea according to the latest ISO (20715: 2023). Generally, aroma plays a more important role than other factors during the sense-based quality evaluation of tea products and has an important effect on tea manufacture and consumption; thus, volatile analysis has become the focus of tea chemistry. According to the fermentation level, tea is divided simply into two types: fermented and unfermented tea. Green tea is representative of unfermented teas and mainly exhibits Qingxiang and chestnut-like aromas. To date, the chemistry characteristics of green tea have been explored deeply, clarifying the difference between cultivars, manufacturing techniques, and aroma types and revealing the change mechanism of key components during tea manufacturing [1,2,3]. For fermented teas, black tea is regarded as a typical example and demonstrates a sweet/floral aroma, and its key volatile compounds differ greatly from those of green tea. The fresh leaves of green tea are inconsistent with black tea and there is a more obvious difference between their key components [4,5,6]. Such a phenomenon also exists between other teas, so appropriate material should be selected in order to analyze the differences between many types of tea products.

In recent years, a variety of teas have been investigated, including cultivars such as *Camellia sinensis* (L.) Kuntze ‘Tieguanyin’, *C. sinensis* ‘Huangdan’ [7], *C. sinensis* ‘Xingrenxiang’ [8], and *C. sinensis* ‘Jinguanyin’ [9] that are prepared for oolong tea, black tea, and white tea. These tea plants have the potential to make highly aromatic teas but have fewer trichomes, so this white tea sample lacks Haoxiang characteristics [8] and the other important features. Some cultivars with many trichomes, such as *Camellia sinensis* (L.) Kuntze ‘Fuyun 6’, *C. sinensis* ‘Huacha 1’, *C. sinensis* ‘Huacha 2’, and *C. sinensis* ‘Fuan Dabai’ (a good tea plant for breeding), have successfully produced types of green tea, black tea, oolong tea, and white tea. The differences in the water-soluble components between their products have been discovered [7,9], but their importance has changed in the minds of consumers and the volatile compounds in them remain unclear. As is known, the aroma quality of all teas relies on volatile compounds. Hundreds of volatile components have been identified so far using gas chromatography–mass spectrometry (GC-MS), along with headspace solid-phase microextraction (HS-SPME), simultaneous distillation extraction (SDE), stir bar sorptive extraction (SBSE), and solvent-assisted flavor evaporation (SAFE) and their constituents and contents vary among different teas. However, some components with low concentrations cannot be detected using GC-MS and remain unexposed. Fortunately, a new type of GC-IMS with a low detection limit has been applied in the field of food science and has received many positive comments [10,11]. The approach has also been shown to have advantages in the volatile analysis of tea samples [12,13,14], so it was selected for use in this study to further explore the aroma chemistry of tea. Based on the abovementioned factors, the tea plant *C. sinensis* ‘Zhongcha 111’ was chosen as our material: this cultivar has many trichomes, is bred from the offspring of the *C. sinensis* ‘Yungui Daye’, and is growing in tea plantations located in Zhejiang province, China. Meanwhile, *C. sinensis* ‘Zhongcha 111’ is also a good material for making green tea and has been promoted throughout the country [15]. In this work, four types of teas were made with fresh leaves of *C. sinensis* ‘Zhongcha 111’ and the volatile characteristics were analyzed with GC-IMS and multivariate statistics. We hoped to explore: (1) the feasibility of GC-IMS to distinguish between tea samples; (2) the difference of VOCs between tea samples; and (3) the potential of this cultivar and utilization.

## 2. Materials and Methods

### 2.1. Tea Plant Material and Chemicals

*Camellia sinensis* cv *C. sinensis* ‘Zhongcha 111’ grows in the tea plantations of Shengzhou, Zhejiang province, China, located at 29°35′ north latitude and 120°49′ east longitude. The tea plant has been scientifically managed for 7 years since 2016 and its new shoots are harvested at the end of March. In this experiment, the picking standard for the collection of fresh leaves was a bud with one leaf. The internal standard 2-octanol was purchased from Beijing Solarbio Science & Technology Co., Ltd., Beijing, China.

### 2.2. Methods

#### 2.2.1. Tea Sample Manufacture

The fresh leaves of *C. sinensis* ‘Zhongcha 111’ were divided into four parts and turned into green tea, yellow tea, white tea, and black tea using traditional technology. The withering condition of the tea samples was consistent, although the withering time differed between samples. The withering time of green tea was as long as for yellow tea and reached 10 h, and the withering time of white tea reached 64 h and was 46 h longer than that of black tea. The spreading leaves were fixed at 100 °C, and the rolling time of the leaves for green tea and yellow tea was 30 min and for black tea was 1 h. The fermentation time of the rolling leaves for black tea was 4 h under natural conditions. The green tea, yellow tea, and black tea were dried under the same conditions (80–90 °C), and the white tea was dried at a lower temperature (60–70 °C).

#### 2.2.2. Sense-Based Quality Valuation

According to the GB/T 23776-2018 method for sense-based quality evaluation, the tea samples were analyzed by 5 professional tea reviewers (including 3 women and 2 men). Specifically, 3 g of green tea was extracted using 150 mL of boiling water for 4 min, while 3 g of yellow tea, white tea, and black tea were extracted for 5 min. After extraction, the reviewers assessed the aroma quality of each tea quickly and provided scores and suitable comments for the samples.

#### 2.2.3. Analysis of Volatile Compounds

The volatile compounds for the different tea samples were detected using a GC-IMS instrument (Flavourspec^®^, G.A.S, Dortmund, Germany) according to the method described by Yang et al. [14] and modified slightly. Briefly, 0.3 g of tea samples, ground with a mortar and pestle, were placed into a 50 mL centrifuge tube and 30 mL of boiling water and 80 μg of 2-octanol were added. The samples were heated for 5 min at 70 °C. The extraction was then cooled and vigorously mixed. Then, 5 mL of the supernatants was put into 20 mL sample bottles and subsequently incubated at 70 °C for 20 min at an agitation speed of 500 rpm. Then, 500 μL of the headspace was automatically injected into the inlet via a heated syringe at 65 °C. The chromatographic separation was performed on an MXT-5 capillary column (15 m × 0.53 mm × 1 μm, Restek, Beijing, China). High-purity nitrogen was employed as a carrier gas using the following programmed flow: increased 2 mL/min for 2 min, reaching 100 mL/min within 18 min, and held for 10 min. The drift tube was 98.0 mm long, with a drift gas flow rate of 150 mL/min. The temperatures of the column and drift tube were kept at 60 °C and 45 °C, respectively. The procedure was repeated 3 times for every sample.

#### 2.2.4. Data Processing

The volatile compounds of the tea samples were identified with the help of the NIST database and some other studies [3,14]. All chemical analyses were expressed as the mean ± SD. A heatmap was created at https://www.chiplot.online/, accessed on 23 April 2023, and multivariate statistical analysis was performed using Metaboanalyst 5.0 software. PLS-DA was used to find the differences in the total metabolites among the 12 samples.

## 3. Results and Discussion

### 3.1. Profiles of the Volatile Compounds of Tea Samples

According to shopping behaviors and habits, green tea with a chestnut-like aroma and corn-aroma yellow tea usually gain high recognition. In this test, all of the samples made with fresh leaves of *C. sinensis* ‘Zhongcha 111’ showed good sense-based qualities, and their aroma scores were above 90 (Table S1), so they acted as excellent representatives of green, yellow, white, and black teas. The samples have sweet odors. The concentrations of the green, yellow and black teas was richer than that of white tea.

The volatile compounds (VOCs) of these samples were detected using HS-GC-IMS and the ensuing data were depicted in a 2D topographical visualization (Figure S1). Figure S1 shows that the topographic plots of volatile fingerprints for green tea were similar to yellow tea and different from white tea and black tea. Unfortunately, it was a challenge to quickly find their nuances, especially between white and black tea. A total of 104 VOCs were detected in our samples, each with a carbon number less than 15 (Table S2). The total content of VOCs for white tea reached 1400.9 ± 226.8 μg/g, which was 33.1%, 55.3%, and 66.2% higher than that of the black, yellow, and green teas, respectively. Among them, 93 components including 16 dimers were identified, the proportions of which ranged from 85.9% to 92.4%. These known compounds included 19 alcohols, 25 aldehydes, 12 ketones, 14 esters, and 6 olefins. There was a wider variety of aldehydes than other compounds, with an average content of 232.5 μg/g. Alcohols were superior to aldehydes at less than 200 μg/g [16,17] but accounted for 14.2% more than esters. The average content of olefins, sulfur compounds, nitrogenous heterocyclic compounds, acids, alkanes, anilines, ethers, oxygen heterocyclics, and nitriles was less than 100 μg/g.

**Figure 1:**
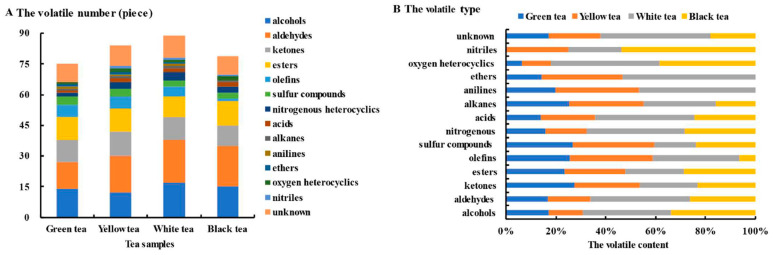
Number and content of volatile compounds in different tea samples. (**A**) Number of volatile compounds, (**B**) proportion of volatile compounds.

The number and content of volatile compounds differed among the tea types. The number of volatile compounds ranged from 75 to 89 (Figure 1A), and the number of unknown compounds in white tea was significant. In white tea, 78 components were identified via GC-IMS, which was a higher number than that found in the other samples. Furthermore, aldehydes and alcohols showed good superiority in terms of number and content among our samples (Figure 1B); this result is consistent with that of Luo et al. [18]. In this study, the aldehyde content of white tea was 53.2% higher than that of black tea, inconsistent with the findings of Wang et al. [19] and Chen et al. [17]; however, its alcohol content was close to that of black tea. The alcohol content of black tea was 99.8% and 144.0% higher than that of green tea and yellow tea, respectively. The aldehyde content of green tea was close to that of yellow tea, with only five more aldehydes being identified. The number of ketones was similar to the number of esters, but the ketone content of the fermented teas (white and black) was lower than that of the non-fermented ones (green and yellow), which was a result of the fermentation process. The ketone content of white tea was close to that of black tea, accounting for the withering technology used. There was one more sulfur compound found in the fermented teas than in the non-fermented types, but the content of these compounds in yellow tea was the largest and reached 70.4 μg/g, indirectly highlighting the importance of sealed yellowing. The number of acids, alkanes, anilines, ethers, and oxygen heterocyclics was low in all samples, and in green tea only one was identified. Both anilines and ethers in black tea were unidentified, and nitriles were not determined in green tea.

Non-volatile dimers such as dimeric catechins are detected via UPLC-MS [20,21], and the volatile dimers of teas are reported only from GC-IMS analysis [3,10], though GC-MS is often applied to analyze the volatile compounds. As mentioned before, 16 dimers were identified from the tea products of *C. sinensis* ‘Zhongcha 111’, and the order of their contents was white tea > yellow tea > green tea > black tea. The number of dimers in yellow tea reached 14 and was equal to white tea. 2-Pentanone, 3-methyl-2-butenal, ethyl 2-methylpentanoate, and their dimers were detected in the four types of tea; in white tea, the content of these dimers was large, while in green tea it was small. In yellow tea and black tea, the contents of the 3-methyl-2-butenal dimer and ethyl 2-methylpentanoate dimers was lower than that of their monomers. Benzaldehyde dimer and linalool oxide dimer, similar to their monomers, were only found in white tea and black tea, suggesting that this may be related to fermentation techniques. 2-Methylpropanal and 3-methyl butanal with high OAV were key volatile components in green tea and black tea [3,22], but the dimers of 2-hexanone, 2-methylpropanal, and 3-methyl butanal were not determined only in black tea, the underlying cause of which was unclear.

### 3.2. Four Types of Teas Distinguished according to Volatile Compound Content

**Figure 2:**
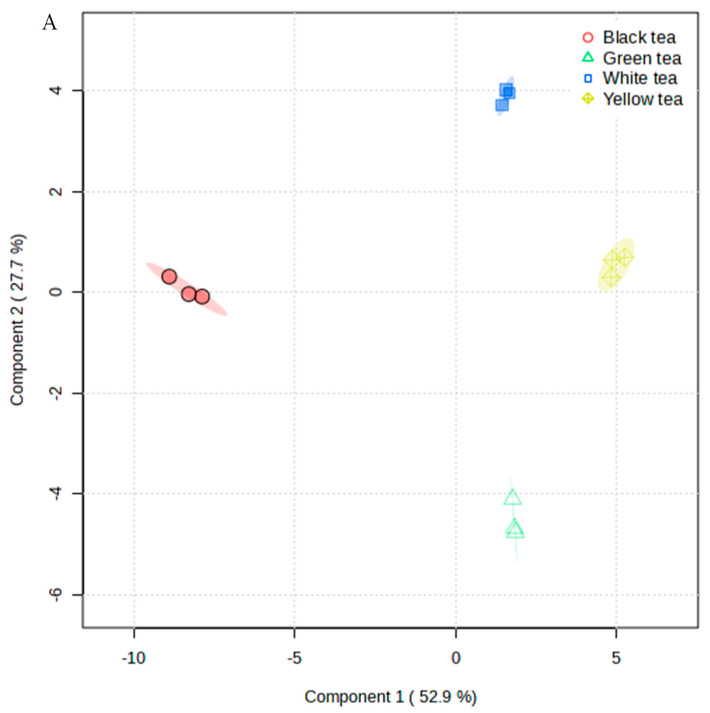

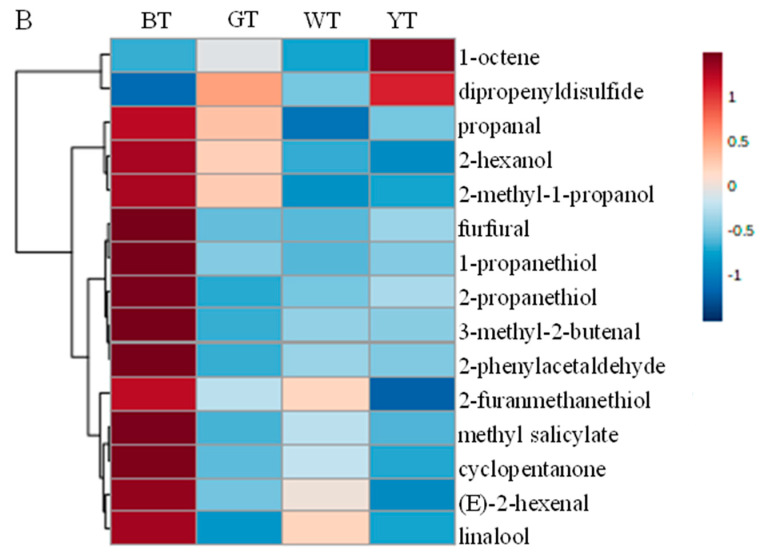
Multiple statistical analysis of volatile compounds between samples. (**A**) PLS-DA of volatile compounds; (**B**) volatile compounds with VIP > 1.5.

Based on the volatile compounds found in the samples, the four types of teas (12 samples) could be differentiated clearly by PLS-DA (Figure 2A), and two components added up to 80.6% (R^2^ = 0.92, Q^2^ = 0.86). Green tea was separated at an area different from the others, and white tea was nearer to yellow tea than black tea (close to the origin). The 15 important variables in the projection (VIP) that contributed to the group separation were 1-octene, dipropenyldisulfide, propanal, 2-hexanol, 2-methyl-1-propanol, furfural, 1-propanethiol, 2-propanethiol, 3-methyl-2-butenal, 2-phenylacetaldehyde, 2-furanmethanethiol, methyl salicylate, cyclopentanone, (E)-2-hexenal, and linalool (Figure 2B). Dipropenyldisulfide, known as diallyl disulfide, improves the anti-obesity effect of green tea in high-fat/high-sucrose-diet-induced obesity [23]; the highest content of this compound was in yellow tea. Except for dipropenyldisulfide and 1-octene, the contents of the other compounds in yellow tea were relatively low, but they were high in black tea. Furthermore, green tea’s low content of furfural, 1-propanethiol, 2-propanethiol, 3-methyl-2-butenal, 2-phenylacetaldehyde, methyl salicylate, and cyclopentanone was similar to that of yellow tea and white tea. Finally, the contents of 2-furanmethanethiol, (E)-2-hexenal, and linalool in white tea were greater than that in green tea and yellow tea.

### 3.3. Comparison of Volatile Compounds among Four Types of Teas

#### 3.3.1. Main Components

The main components of every type of tea were at a different level (Table S2). In green tea, 2,3-pentanedione, 3-methyl butanal dimer, and 2-pentanone were present at higher amounts than the other compounds, while in yellow tea 3-methyl-2-butenal, pentanal, and mesityl oxide were the most prevalent. Furthermore, only the content of mesityl oxide was over 20 μg/g. For white tea, not only did benzaldehyde, ethyl butanoate, and (Z)-hex-2-enal have a good accumulation, adding up to a total 67.6 μg/g, but also beta-pinene, butanoic acid, (Z)-4-heptenal, linalool oxide, (Z)-4-heptenal dimer, 2-methyl-2-pentenal, and heptanal had contents of more than 20 μg/g. For black tea, the top three compounds were hexyl acetate, furfural and 3-methyl butanal, and their contents were more than that of white tea. Therefore, fatty acid derivatives such as pentanal, ethyl butanoate, (Z)-hex-2-enal, butanoic acid, and so on, have an important role in aroma quality, which is consistent with Guo et al. [24].

The contents of 1,4-cineole and 3-methylbutanoic acid ethyl ester, only detected in white tea, were more than 20 μg/g. Except for these compounds, some components such as 2-isopropyl-3-methoxy pyrazine, 3-methyl-3-buten-1-ol, methyl salicylate, and trans-2-pentenal were very low in non-fermentation tea. Few (E)-2-hexenal, 2-methyl-1-butanol, 1-nonanal, 2-furanmethanethiol, 2-hexanol, ethyl butanoate, and heptanal molecules were accumulated in yellow tea. The number of VOCs exceeding 20 μg/g in white tea was the largest among the samples, whereas in yellow tea it was the smallest. Compared with white tea, black tea had a deep fermentation that might make the amount of some VOCs decrease, whereas green tea had a short time spreading that might limit to the volatile formation and conversion. Therefore, the volatile characteristics of the tea samples were influenced by the manufacturing technology.

#### 3.3.2. Common Components

**Figure 3:**
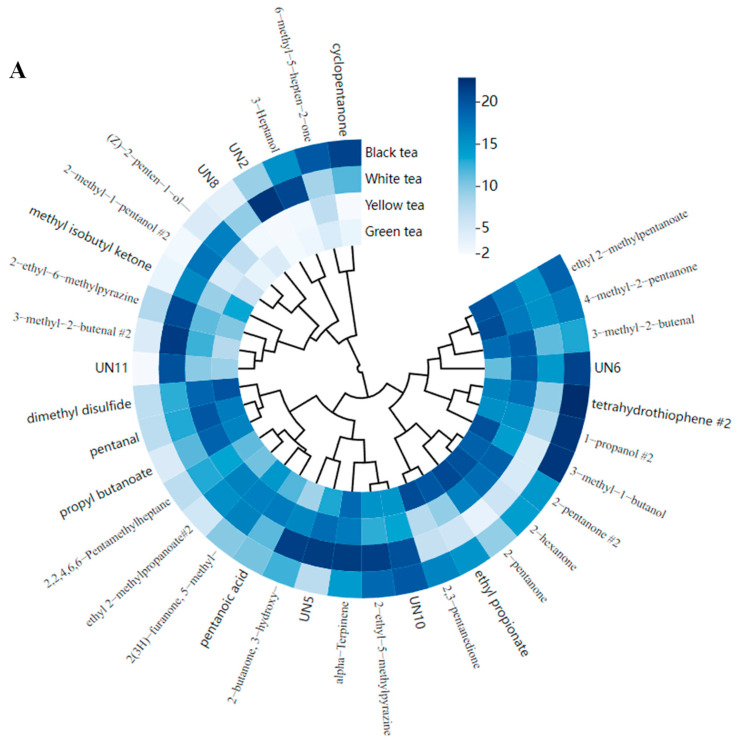

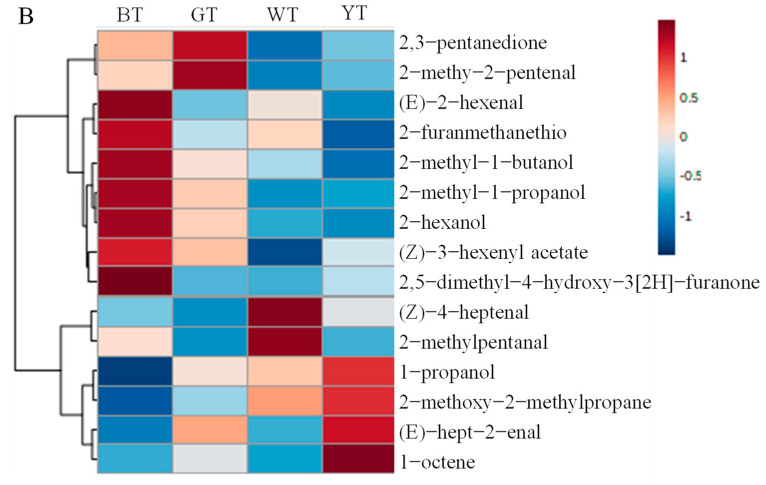
Difference in volatile compounds in four types of tea. (**A**) Common components; (**B**) differential components.

The four types of tea were made with the fresh leaves of *C. sinensis* ‘Zhongcha 111’. There were 35 common components (Figure 3A), including 29 identified ones with a total content reaching 386.0 μg/g, which was 6.6-fold higher than the five unidentified components (Table S2). The content of common compounds in white tea was high; the content of unidentified compounds was also high. The average content of alpha-terpinene was the highest among the 29 identified compounds and 129.8% higher than the lowest (2-methyl-1-pentanol dimer). The content of common components varied according to the tea type. Besides the unknown compounds, the contents of 2-hexanone, 2-pentanone, 3-methyl-1-butanol, 4-methyl-2-pentanone, dimethyl disulfide, and ethyl 2-methylpentanoate were high in green tea, and the total content reached 122.3 μg/g, accounting for 30.1% of the common components. However, in white tea, the amount of these compounds was low, especially 2-hexanone and 2-pentanone.

The withering time of white tea was the longest among the four types of tea, and its contents of 3-hydroxy-2-butanone, 2-ethyl-5-methylpyrazine, 2-ethyl-6-methylpyrazine, 3-heptanol, 3-methyl-2-butenal dimer, and alpha-terpinene were relatively high. Their total content was 128.9 μg/g in white tea, accounting for 33.2% of the common components. The content of 2-ethyl-5-methylpyrazine, belonging to the group of nitrogenous heterocyclic compounds, ranged from 12.7 μg/g to 21.6 μg/g and was higher than the content of 2-ethyl-6-methylpyrazine. The difference between the contents of 2-ethyl-5-methylpyrazine and 2-ethyl-6-methylpyrazine was largest in black tea. The amount of alpha-terpinene in white tea was close to that of 2-ethyl-5-methylpyrazine, but it was 4.2 μg/g lower than that in black tea. The amount of 3-heptanol in white tea reached 21.0 μg/g and was 7.5-fold, 8.3-fold, and 1.4-fold greater than that in green, yellow, and black teas, respectively.

In black tea, the contents of common compounds such as 1-propanol dimer, 3-methyl-1-butanol, cyclopentanone, and tetrahydrothiophene dimer were relatively high and over 20.0 μg/g. 3-Methyl-1-butanol also showed a good accumulation in black tea, which was similar to that in green tea, reaching 22.2 μg/g. The cyclopentanone content in black tea was higher than the average, but in green, white, and yellow teas it was below the average. Compared with the other samples, the content of common compounds in yellow tea was relatively low, with pentanal being present in the largest amounts but still constituting under 20.0 μg/g.

#### 3.3.3. Differential Components

The above samples were manufactured using different techniques and their aromatic characteristics differed greatly. A one-way analysis of variance (ANOVA) indicated that there were 96 differential components between the tea samples (*p* < 0.05). The top 15 components were 2,3-pentanedione, 2-methy-2-pentenal, (E)-2-hexenal, 2-furanmethanethio, 2-methyl-1-butanol, 2-methyl-1-propanol, 2-hexanol, (Z)-3-hexenyl acetate, 2,5-dimethyl-4-hydroxy-3[2H]-furanone, (Z)-4-heptenal, 2-methylpentanal, 1-propanol, 2-methoxy-2-methyl, (E)-hept-2-enal, and 1-octene (Figure 3B). This finding was inconsistent with the results of the PLS-DA, but (E)-2-hexenal, 2-furanmethanethio, 2-hexanol, and 1-octene were also selected as differential components. In yellow tea, (E)-2-hexenal, 2-furanmethanethio, and 2-hexanol were undetected (Table S2), perhaps resulting from yellowing techniques [25]. We found that 1-octene-3-ol was a key volatile compound in several kinds of tea [26,27,28] but 1-octene containing the same number of carbon atoms was not detected in white tea or black tea, suggesting that this might be related to fermentation technology.

The average content of 2,3-pentanedione was lower than that of 2-methy-2-pentenal, but they showed the same order of green tea > black tea > yellow tea > white tea. (E)-2-Hexenal, 2-furanmethanethio, 2-methyl-1-butanol, 2-methyl-1-propanol, 2-hexanol, (Z)-3-hexenyl acetate, and 2,5-dimethyl-4-hydroxy-3[2H]-furanone were rich in black tea, while (Z)-4-heptenal and 2-methylpentanal were abundant in white tea. In contrast, in yellow tea, 1-propanol, 2-methoxy-2-methylpropane, (E)-hept-2-enal, and 1-octene accumulated easily.

## 4. Conclusions

*C. sinensis* ‘Zhongcha 111’ has, once again, been verified as a favorable tea plant. Its white tea was shown to contain more aldehydes than the other samples when HS-GC-IMS was applied to determine the volatile compounds. Four types of samples were distinguished according to the identified compounds and, using the PLS-DA approach, 15 key compounds (VIP > 1.5) including differential components were detected. Moreover, 29 common components including 3-methyl-2-butenal and dimethyl disulfide were also identified as differential compounds, varying according to the manufacturing technology. The findings indicate that HS-GC-IMS is an appropriate method for use in differentiating tea samples from the same material.

## Data Availability

The original contributions presented in the study are included in the article/Supplementary Material, further inquiries can be directed to the corresponding authors.

## Supplementary Materials

The following supporting information can be downloaded at: https://www.mdpi.com/article/10.3390/foods13132043/s1, Figure S1: Fresh leaves of *C. sinensis* ‘Zhongcha 111’, Figure S2: The two-dimensional spectrum of VOCs from the tea samples, Table S1: The aroma quality of the tea samples, Table S2: The content of volatile compounds in samples.
