# Supplementary material for: Comparison of Volatile Compounds among Four Types of Teas Analyzed Using Gas Chromatography–Ion Mobility Spectrometry
Source: Foods. 2024 Jun 27;13(13):2043. doi: 10.3390/foods13132043 (PMC11241802; doi:10.3390/foods13132043)
Supplement: Supplementary file 1 [file foods-13-02043-s001.zip › foods-3060760-supplementary.pdf]

Table S1 The aroma quality of tea samples

Samples	Comments	Score
**Green tea**	rich, sweet and high chestnut-aroma	94.0
**Yellow tea**	rich, sweet and high corn-aroma	94.0
**White tea**	fresh, sweet aroma	91.0
**Black tea**	rich, sweet aroma	93.0

Table S2 The content of volatile compounds in samples

Compoud(μg/g)	Green tea	Yellow tea	White tea	Black tea
**(E)-2-hexenal**	4.1±0.2	nd	16.9±2.5	22.7±3.0
**(Z)-4-heptenal**	nd	2.4±0.3	22.4±3.6	nd
**(E)-2-Pentenal**	nd	3.2±0.1	20.6±4.3	10.3±1.0
**(E)-hept-2-enal**	13.5±0.2	18.4±1.3	7.7±1.5	nd
**(Z)-2-Penten-1-ol**	3.2±0.1	6.8±0.4	16.6±3.0	4.6±0.8
**(Z)-3-hexenyl acetate**	9.3±0.2	6.6±0.6	nd	15.0±1.7
**(Z)-4-heptenal dimer**	nd	4.0±0.5	22.0±3.8	2.4±0.3
**1,4-Cineole**	nd	nd	21.0±3.2	nd
**1-butanol 2-methyl**	6.2±0.5	nd	9.0±2.0	12.8±1.0
**1-nonanal**	4.7±0.4	nd	11.6±2.1	5.4±0.5
**1-Octene**	6.5±0.2	18.8±2.2	nd	nd
**1-Propanethiol**	1.2±0.1	1.1±0.3	nd	17.4±0.9
**1-Propanol**	9.0±0.3	13.5±1.9	20.4±4.3	nd
**1-Propanol dimer**	15.3±0.7	15.3±2.5	7.9±1.3	22.3±2.3
**1-Propanol, 2-methyl-**	7.9±0.1	nd	nd	16.3±2.3
**2(3H)-Furanone, 5-methyl-**	14.6±0.3	16.6±1.6	16.4±1.9	9.8±1.8
**2,2,4,6,6-Pentamethylheptane**	11.3±0.7	13.5±0.9	13.0±2.7	7.1±1.3
**2,2-Dimethyl-3-methylenebicyclo[2.2.1]heptane**	5.4±0.3	6.1±0.5	10.6±2.0	nd
**2,3-pentanedione**	20.7±0.2	7.5±0.9	6.5±0.2	16.2±1.9
**2,5-dimethyl-4-hydroxy- 3[2H]-furanone**	nd	2.6±0.1	nd	14.7±1.2
**2-acetylfuran**	3.1±0.9	3.0±0.9	nd	3.8±0.4
**2-Butanone, 3-hydroxy-**	8.6±0.6	16.0±1.3	21.6±2.9	12.3±1.5
**2-ethyl-5-methylpyrazine**	15.0±0.3	12.8±1.4	21.6±4.0	18.4±2.8
**2-Ethyl-5-methylpyrazine dimer**	nd	2.3±0.5	2.8±0.7	nd
**2-Ethyl-6-methylpyrazine**	10.1±1.0	11.3±1.3	20.9±4.4	7.8±1.1
**2-furanmethanethiol**	8.0±0.3	nd	21.3±4.0	19.8±2.3
**2-Heptanone**	nd	6.2±0.6	19.3±3.2	nd
**2-Hexanol**	10.8±0.2	nd	5.3±0.7	22.5±2.5
**2-Hexanone**	20.3±0.7	18.1±2.9	5.9±1.6	14.0±1.8
**2-Hexanone dimer**	11.8±0.2	14.4±2.4	21.4±4.2	nd
**2-Isopropyl-3-methoxy pyrazine**	nd	nd	17.4±2.4	18.5±1.4
**2-Methoxy-2-methylpropane**	5.6±0.7	12.6±0.8	20.7±4.3	nd
**2-Methyl propyl acetate**	12.0±0.3	16.4±1.6	21.4±4.4	nd
**2-Methyl-1-pentanol**	3.4±0.6	3.7±0.3	21.2±2.3	nd

**2-Methyl-1-pentanol dimer**	6.2±0.2	5.0±0.1	17.5±2.6	2.5±0.5
**2-Methyl-2-pentenal**	10.5±0.4	10.7±1.0	22.1±3.8	nd
**2-Methyl-2-pentenal dimer**	12.4±2.2	2.2±0.0	nd	7.3±1.6
**2-Methylpentanal**	nd	1.1±0.2	21.9±3.5	5.3±0.6
**2-Methylpropanal**	18.8±0.9	9.4±1.5	nd	16.5±1.7
**2-Methylpropanal dimer**	13.6±0.3	14.4±1.7	19.9±3.7	nd
**2-Pentanone**	20.9±0.4	16.6±1.8	3.4±0.1	9.3±1.0
**2-Pentanone dimer**	19.1±0.6	19.0±1.9	4.9±0.6	14.6±2.0
**2-phenylacetaldehyde**	nd	2.1±0.2	5.9±1.0	22.8±2.4
**2-Propanethiol**	nd	3.9±0.5	4.5±0.7	22.0±2.5
**3-Butenenitrile**	nd	7.4±0.8	6.3±1.2	15.8±1.6
**3-Heptanol**	2.8±0.2	2.5±0.2	21.0±3.0	15.2±1.5
**3-Methyl butanal**	nd	2.6±0.3	6.0±0.9	23.9±2.6
**3-Methyl-1-butanol**	20.3±0.4	14.0±1.9	4.8±1.1	22.2±1.4
**3-Methyl-2-butenal**	18.2±1.3	19.6±2.0	11.3±1.2	13.2±0.7
**3-Methyl-2-butenal dimer**	7.6±1.4	12.4±1.2	21.8±3.2	4.5±0.8
**3-Methyl-3-buten-1-ol**	nd	nd±	14.1±2.1	6.7±0.6
**3-methyl butanal dimer**	20.9±0.4	19.2±2.4	17.5±2.6	nd
**4-methyl-2-pentanone**	20.6±0.5	17.1±2.2	15.2±2.4	17.0±1.6
**alpha-Pinene**	13.1±0.4	15.6±1.6	21.9±3.9	nd
**alpha-Terpinene**	18.3±1.6	17.1±0.4	21.9±3.9	14.3±1.3
**Benzaldehyde**	nd	nd	22.5±3.5	10.2±1.8
**Benzaldehyde dimer**	nd	nd	21.7±3.8	7.9±0.9
**beta-Pinene**	9.6±0.6	12.6±1.3	22.2±3.8	nd
**Butanoic acid**	nd	1.4±0.1	22.3±3.3	9.9±1.3
**Butanoic acid, 3-methyl-, ethyl ester**	nd	nd	21.4±3.3	nd
**Butanoic acid, 3-methyl-, ethyl ester dimer**	14.2±0.7	12.8±2.0	nd	23.3±2.0
**cyclopentanone**	3.0±0.3	2.0±0.3	11.8±2.1	21.3±3.1
**Dimethyl disulfide**	20.0±1.1	18.7±2.2	12.8±1.4	7.0±0.9
**dipropenyldisulfide (diallyl disulfide)**	15.5±0.7	19.6±2.5	14.1±2.7	nd
**ethyl 2-methylpentanoate**	20.1±0.8	17.3±2.7	15.0±2.5	18.9±1.7
**Ethyl 2-methylpropanoate dimer**	10.7±0.5	16.4±2.3	15.3±2.9	5.4±0.8
**Ethyl 3-methylbutanoate**	19.4±1.0	19.3±2.9	nd	11.5±2.0
**Ethyl Acetate**	nd	6.0±1.0	10.4±1.8	21.0±2.7
**Ethyl butanoate**	1.8±0.0	nd	22.5±3.5	12.1±1.4
**ethyl propionate**	19.8±1.3	9.4±0.1	5.7±1.3	14.9±2.5
**Furfural**	nd	2.4±0.3	nd	23.7±2.7
**Heptanal**	3.6±0.1	nd	22.2±3.7	14.7±2.2
**(Z)-hex-2-enal**	nd	nd	22.6±3.5	15.8±1.7
**Hexyl acetate**	10.0±0.3	14.6±2.4	nd	23.4±2.1
**Isoprene**	4.9±0.6	4.8±0.6	2.1±0.0	nd
**Linalool**	nd	1.0±0.1	19.4±3.2	22.9±2.4
**Linalool oxide**	nd	nd	22.0 3.8	17.9±0.7
**Linalool oxide dimer**	nd 0.0	nd 0.0	19.9 1.8	19.4±2.1

**Mesityl oxide**	15.3	0.3	20.1	2.5	0.0	0.0	7.2	0.9
**Methyl isobutyl ketone**	13.4	1.2	9.1	1.4	15.9	2.8	3.1	1.0
**Methyl salicylate**	nd	0.0	nd	0.0	9.1	1.5	20.7	1.9
**6-methyl-5-hepten-2-one**	4.7	0.7	6.9	1.4	8.5	1.6	19.6	2.2
**N,N-diethylethanamine**	9.1	1.2	15.4	2.5	21.5	3.9	nd	0.0
**pentan-1-ol**	10.0	0.6	13.9	1.8	9.6	1.0	nd	0.0
**Pentanal**	16.9	0.9	19.8	2.1	13.1	2.2	7.1	1.1
**Pentanal dimer**	nd	0.0	10.0	0.1	20.5	2.2	2.6	0.4
**Pentanoic acid**	11.8	1.0	16.9	0.6	11.5	0.5	10.5	1.2
**Propanal**	12.0	0.1	5.1	0.9	nd	0.0	22.1	3.0
**Propyl butanoate**	16.3	0.1	18.9	2.4	11.6	1.8	4.6	1.1
**tert-butanol**	18.0	0.5	19.4	1.9	20.7	3.8	nd	0.0
**Tetrahydrothiophene**	5.4	0.4	14.2	1.4	nd	0.0	20.6	3.0
**Tetrahydrothiophene dimer**	16.4	0.8	17.9	1.4	9.4	1.6	22.9	2.2
**trans-2-pentenal**	nd	0.0	nd	0.0	21.9	3.5	3.7	0.4
**nd1**	4.8	0.8	7.6	0.8	9.7	2.3	nd	0.0
**nd2**	2.2	0.1	2.1	0.0	22.3	3.4	9.1	0.9
**nd3**	8.1	0.7	13.9	1.6	21.1	4.5	nd	0.0
**nd4**	nd	0.0	1.5	0.3	16.3	3.2	3.6	0.7
**nd5**	13.2	1.0	18.1	1.4	21.9	3.9	7.2	1.3
**nd6**	11.1	1.3	19.5	1.9	14.5	1.0	21.3	2.7
**nd7**	8.3	1.0	nd	0.0	19.6	1.6	8.6	0.9
**nd8**	4.5	0.3	2.5	0.3	9.4	5.9	3.8	0.5
**nd9**	nd	0.0	2.9	0.2	21.4	4.2	4.2	0.4
**nd10**	14.7	1.5	13.5	1.8	20.3	5.5	19.6	3.8
**nd11**	9.0	0.9	9.7	1.0	20.2	2.0	2.1	0.2
